# Phase response curves of subthalamic neurons: experimental measurement and theoretical prediction

**DOI:** 10.1186/1471-2202-12-S1-P370

**Published:** 2011-07-18

**Authors:** Michael A Farries, Charles J Wilson

**Affiliations:** 1 Department of Biology, University of Texas San Antonio, San Antonio, TX 78249, USA

## 

The subthalamic nucleus (STN) is an autonomously active population of glutamatergic neurons that occupies a pivotal location within the basal ganglia, receiving direct cortical input and innervating the globus pallidus and substantia nigra. As intrinsically oscillating neurons, STN cells may be suitable candidates for reduction to phase models, where the state of each neuron is represented by a single number (phase) and their response to synaptic input is given by infinitesimal phase response curves (iPRCs). Phase models are analytically tractable and assist the study of large neural populations, but whether they can describe the behavior of STN cells with sufficient accuracy is an open question. We addressed this issue by measuring the iPRCs of STN cells in brain slices, using both glutamatergic synaptic input and brief current injection. We found that iPRCs measured synaptically were relatively insensitive to the size of the EPSP used to measure them, and that iPRCs measured with current pulses were the same regardless of the polarity of the current, at least during the first 60% of the oscillation cycle; both observations suggest that iPRCs might accurately describe the response of STN cells to finite inputs. However, we also found that iPRCs measured synaptically and by current injection were dramatically different, with current pulse iPRCs giving much less phase shift for a given stimulus amplitude.

We developed and analyzed a biophysical model of STN cells, to better understand the nature of subthalamic iPRCs. This model produced an iPRC that closely resembled those we measured experimentally. Observing that this model consisted mainly of kinetically fast voltage-dependent currents and AHP currents that can be treated as a function of time since the last spike, we explored the iPRCs of cells whose conductances are direct functions of voltage. Ignoring the spike itself by assuming that it is triggered at a certain threshold potential followed by return (2 ms later) to resetting potential, we found that the iPRC of such cells is a simple function of their IV curves and that their iPRC is valid for arbitrarily large currents. The contribution of AHP currents could therefore be covered by treating them as applied currents acting through this IV curve-based iPRC. Following this approach, we developed a method for deriving the iPRC of any cell well described by a combination of fast voltage-dependent currents and time-dependent AHP currents, covering a wide range of cell types. We were also able to estimate experimentally the IV curves and AHP currents of STN cells in which we had empirically determined the synaptic and current pulse iPRCs, allowing us to compare theoretically predicted iPRCs to experimentally measured iPRCs. We found that the theoretically predicted PRC approximated the synaptic--but not the current pulse--iPRC. We hypothesize that somatically injected charge slowly trickles into distal dendrites, reducing its effectiveness at advancing the phase, whereas synaptically delivered charge comes from precharged dendrites. We conclude that current injection is not an effective method for measuring iPRCs in dendritic neurons.

